# Evaluation of centers for information and support combining formal and informal care for patients with cancer: a systematic review of the literature

**DOI:** 10.1007/s00520-022-07047-w

**Published:** 2022-04-13

**Authors:** Helen P. A. Driessen, Leonieke W. Kranenburg, Karin C. D. van der Rijt, Evi M. Bakker, Jan J. van Busschbach, Lea J. Jabbarian, Wichor M. Bramer, Erna J. Elfrink

**Affiliations:** 1 Department of Psychiatry, Erasmus MC - University Medical Center Rotterdam, P.O. Box 2040, 3000 CA, Rotterdam, The Netherlands; 2 Department of Medical Oncology, Erasmus MC Cancer Institute, University Medical Center Rotterdam, Rotterdam, The Netherlands; 3 Department of Public Health, Erasmus MC - University Medical Center Rotterdam, Rotterdam, The Netherlands; 4 Medical Library, Erasmus MC - University Medical Center Rotterdam, Rotterdam, The Netherlands; 5 Erasmus MC Cancer Institute Rotterdam, Rotterdam, The Netherlands

**Keywords:** Neoplasms, Patient experience, Personal satisfaction, Health personnel, Patient care, Social support

## Abstract

**Purpose:**

Clear information and supportive care are necessary for patients with cancer to effectively manage their condition. Traditionally, healthcare professionals offer information and support via the so-called *formal* care route. In addition, research has found favorable effects of *informal* care provided by volunteer programs and informal “walk-in support centers.” Less research has been done on initiatives that combine formal and complementary informal supportive care for patients with cancer. This systematic literature study aimed to discover (1) which types of initiatives are described in the literature, (2) what type of care they offer, and (3). how they are evaluated in terms of outcome measures.

**Methods:**

We performed a systematic literature search of MEDLINE, Embase, PsycINFO, and CINAHL. Studies were included if the collaboration between one type of formal care together with one type of informal care was explicitly mentioned in the article. The search was not restricted to a specific cancer type.

**Results:**

A total of 4869 records were retrieved and 18 studies were included. In most studies, the care provided consisted of emotional support for, and/or providing information to, patients and their families. Initiatives were evaluated with interviews and questionnaires. Patients with cancer reported that they were satisfied with the care offered, including information, social and emotional support, help with activities of daily living, and family-related issues. Volunteers reported that visits they made were experienced positive and rewarding and the volunteers were confident about their contribution to general healthcare. Some negative experiences were reported by volunteers, e.g., interference of their own cancer diagnosis with volunteer work. The importance of proper training was stressed.

**Conclusions:**

Initiatives combining formal and informal supportive care hold the potential of added value in terms of providing emotional support for, and providing information to, patients with cancer. Support and specific training for volunteers can be viewed as success factors in the involvement of volunteers in formal care practices.

**Supplementary Information:**

The online version contains supplementary material available at 10.1007/s00520-022-07047-w.

## Introduction

In uncertain times of cancer diagnosis and treatment, having access to comprehensible and clear information on the disease and its treatment is crucial for patients with cancer [1]. Understanding what is going on and what can be expected helps in coping with the disease. A recent review has shown that informational needs may change over time, and that, in general, informational needs in patients with cancer are not always met [1]. For instance, patients may lack information on how to deal with possible side effects or how to support their recovery or treatment [2]. In addition, patients with cancer are often provided with information at the time of diagnosis, although there is evidence that attention and recollection are severely attenuated when the patient has increased levels of distress, making this not the best of timing [3, 4]. In addition, supportive care that is ‘the prevention and management of the adverse effects of cancer and its treatment’ [5] is important for patients with cancer to manage their condition [6]. Supportive care includes “the management of both physical and psychological symptoms and side effects across the continuum of the cancer experience from diagnosis, through anticancer treatment, to post-treatment care” [5]. For instance, 30% of all patients with cancer suffer from psychological complaints such as adjustment disorders, stress, anxiety, and depression [7]. Moreover, these psychological complaints may lead to psychosocial problems, such as the inability to maintain work or activities of daily living, financial instability, and disturbed family relations [7, 8]. An international survey of patient insights into cancer care found that 69% of the respondents felt the need for psychological and social support during or after their cancer care, and of these, 34% stated it was not available [2]. Supportive care has positive effects on patients’ wellbeing and emotional adjustment to cancer and is associated with fewer psychological complaints [9–17].

Traditionally, patients with cancer receive information and supportive care from healthcare professionals that is through the *formal* care route. In addition, *informal* care can play a role. Informal care can be defined as “unpaid care and may involve a variety of actions, like transport to doctors, social companionship, emotional guidance, or help with arranging professional care” [18]. Informal care is usually provided by relatives and friends, or by volunteers arranged by an institute independent of the hospital, such as informal “walk-in centers” outside the hospital or “peer-support” through an internet community or via fora. Several studies have shown that such informal care, independent of the hospital, is of additional value in coping with the psychosocial impact and practical issues of diagnosis and treatment in patients with cancer and their families [19–25]. Informal supportive care can also be arranged by initiatives that work closely together with institutes or hospitals. The terminology used for such initiatives varies from “information and support centers” [26], “cancer information services” [27], and “cancer navigation services” [28], to programs initiated by National Cancer Societies such as “Reach To Recovery.”

It can be hypothesized that these initiatives combine the benefits of formal care and the benefits of informal care. However, there is no synthesis available of the research undertaken on such initiatives. This review aims to explore what is known about initiatives that combine formal and informal care, in terms of what they offer, and how these initiatives are experienced by, e.g., patients, volunteers, and healthcare staff.

## Methods

### Aim

This systematic literature study is explorative and aimed to learn more about (1) which types of initiatives that combine formal and complementary informal supportive care for patients with cancer are described in the literature, (2) what type of care they offer, and (3) how they are evaluated in terms of outcome measures. In this review, informal care refers to care given by volunteers, for example, volunteers that provide company, a listening ear, creative workshops, and practical assistance. Formal care includes supportive care or the coordination thereof by healthcare professionals. Examples of formal care include care provided by nurses, oncologists, social workers, and psychologists.

### Inclusion and exclusion criteria

Studies were included if they focused on adult patients with cancer and if (1) at least one type of hospital formal care was present (doctor, nurse, hospital psychosocial caregivers) together with (2) at least one type of informal care, such as (peer)volunteers, explicitly named websites or online support programs and non-hospital therapy such as yoga and creative therapy, (3) the collaboration between (1) and (2) was explicitly mentioned/described in the article. Excluded were (i) reviews, (ii) congress abstracts, (iii) articles exclusively on palliative care or (iv) exclusively on children and adolescents. Also excluded were (v) articles wherein informal care was provided by family or friends only. Finally, (vi) intervention studies or studies on the feasibility or the development of certain programs were excluded, unless they were already implemented or about to be implemented. Papers were limited to those written in English.

### Search terms and databases

The main search terms were “Cancer patients,” “Psychosocial care,” and “Health care organization.” There were no restrictions regarding the date of publication. This search was applied to Medline ALL Ovid (1946–present), Embase.com (1971–present), PsycINFO Ovid (1806–present), and CINAHL EBSCOhost (1939–present). The final search was run on July 29, 2021. The full search strategy is reported in Supplementary Table 1. Furthermore, the reference lists of all relevant studies were checked to find additional studies.

### Procedures

One reviewer (HD) screened all article titles, and a second reviewer (either LK, EB, WB, or LJ) reviewed the same set of article titles. If the title of the study appeared to indicate that a combination of formal and informal care could be described, we also reviewed the abstracts. Five authors (HD, LK, EB, WB, LJ) were engaged in the assessment of title and abstract and all titles and abstracts were reviewed by at least two reviewers, and all were assessed by HD. In case of disagreement, the two reviewers aimed to reach a consensus. In case this turned out to be difficult, a third reviewer was involved to come to a conclusion. All included papers were read by two reviewers (HD and EB). Again, in case of disagreement, the two reviewers aimed to reach a consensus. Where this turned out to be difficult, a third reviewer (LK) was involved to come to a conclusion. The quality of qualitative studies was assessed with the aid of the Critical Appraisal Skills Program (CASP) checklist that focuses on qualitative studies.

### Outcomes

We aimed to retrieve information on (1) the organization format, (2) which parties were involved and/or collaborated, e.g., hospitals and organizations, (3) the professions that were involved in providing care and/or coordinating the volunteers, (4) how volunteers were selected and whether they were trained, (5) the setting in which the care was provided, e.g., at the hospital or at the patients’ home settings, (6) the type of care that was provided, and (7) how the initiatives were evaluated.

## Results

**Fig. 1:**
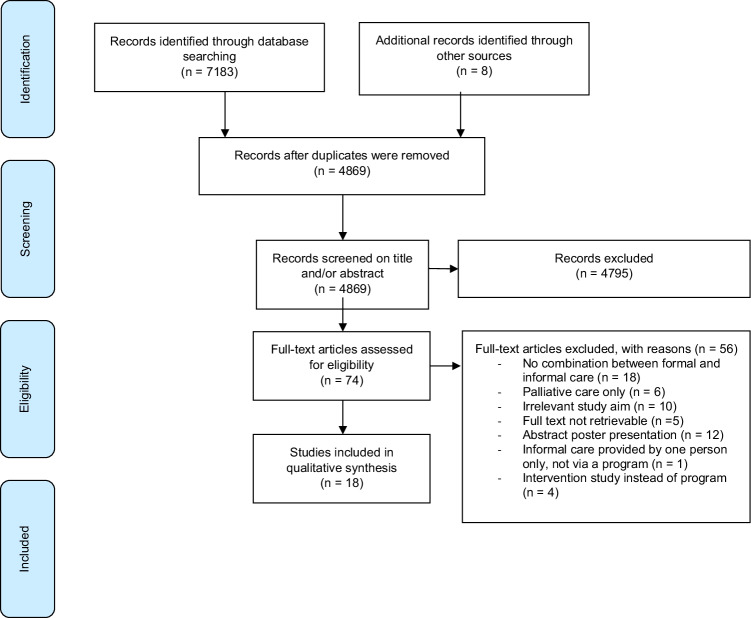
Flowchart of literature search and study selection

A total of 7183 studies were retrieved from the databases, and 8 additional articles were identified through cross-referencing. A total of 4869 studies were eligible for screening after duplicates were removed and eventually 18 studies met the inclusion criteria. Procedures and search results are described in the flowchart of Fig. 1. If the full text was not retrievable via the standard electronic subscriptions of the academic library of the Erasmus Medical Center, then the library sent an international request to other libraries. If that also failed, we personally approached the authors by email. Most exclusions were due to a lack of description of the collaboration between formal and informal care in the professional setting. Furthermore, many excluded articles focused solely on palliative care. The included qualitative studies were assessed with the aid of the CASP checklist. Most qualitative studies described the aim of the research (9/10), the research design (8/10), the recruitment strategy (7/10), and gave a clear statement of findings (7/10). Less frequently mentioned items were ethical issues (5/10), and the relationship between the researcher and the participants (4/10). The results of the quality assessments are summarized in Supplementary Table 2. An overview of the characteristics of the articles included is given in Table 1.

**Table 1:** Characteristics of the included studies

First author	Year of publication	Title	Participants (*N*)		Type of cancer	Start date initiative
Miller [29]	1960	The role of the voluntary health agency in the organization of cancer services	Patients: - Volunteers: -	Family/friends: - Hospital staff: -	All	-
Timothy [30]	1980	The Reach to Recovery Program in America and Europe	Patients: - Volunteers: -	Family/friends: - Hospital staff: -	Breast	1952
Witter [31]	1981	Volunteers in St. Joseph’s Oncology Unit	Patients: - Volunteers: -	Family/friends: - Hospital staff: -	All	-
Garrison [45]	1983	Oncology outreach program: A community and hospital effort	Patients: 31 Volunteers: 20	Family/friends: - Hospital staff: -	All	1983
Fusco-Karmann [32]	1994	Volunteers in hospital and home care: a precious resource	Patients: 246 Volunteers: 127	Family/friends: - Hospital staff: 179	All	1984
Edgar [33]	1996	An oncology volunteer support organization: the benefits and fit within the health care system	Patients: 121 Volunteers: -	Family/friends: 50 Hospital staff: -	All	1981
Jones [44]	2001	More than just a pamphlet: development of an innovative computer-based education program for cancer patients	75 users (patients, family members, and hospital staff)		All	-
Burton [34]	2001	A successful volunteer program showcased during the international year of volunteering: the cancer council NSW’s breast cancer support service	Patients: - Volunteers: 287	Family/friends:- Hospital staff: 21	Breast	1980
Sparks [46]	2001	Local and National uses of a Road to Recovery Evaluation	Patients: 80 Volunteers: 225	Family/friends: - Hospital staff: -	All	-
Turner [35]	2005	Healing Touch for breast cancer patients	Patients: - Volunteers: -	Family/friends: - Hospital staff: -	Breast	2004
Nissim [36]	2009	Transforming the experience of cancer care: a qualitative study of a hospital-based volunteer psychosocial support service	Patients: 15 Volunteers: -	Family/friends: - Hospital staff: -	All	2008
Jasperse [37]	2012	Evaluation of the training and support received by facilitators of a cancer education and support program in New Zealand	Patients: - Volunteers: 17	Family/friends: - Hospital staff: -	All	1991
Moulton [38]	2013	Woman to Woman: A Peer to Peer Support Program for Women With Gynecologic Cancer	Patients: 57 Volunteers: -	Family/friends: - Hospital staff: -	Gynecologic	2003
Lorhan [39]	2015	The role of volunteers at an outpatient cancer center: how do volunteers enhance the patient experience?	Patients: 7 Volunteers: 6	Family/friends: - Hospital staff: 7	All	2008
Loprinzi Brauer [40]	2016	Peer mentorship programs for breast cancer patients	Patients: 69 Volunteers: 31	Family/friends: - Hospital staff: -	Breast	2006
Borregaard [41]	2017	Exchanging narratives—A qualitative study of peer support among surgical lung cancer patients	Patients: 9 Volunteers: 1	Family/friends: - Hospital staff: -	Lung	2014
Ervik [42]	2020	Evaluating a centralized cancer support center in the remote region of Northern Norway	Patients: 181 Volunteers: -	Family/friends: - Hospital staff: -	All	-
Pitcher [43]	2021	‘Bridge of Support’: evaluation of an acute care peer support model for women with breast or a gynecological cancer	Patients: 50 Volunteers: 3	Family/friends: - Hospital staff: 21	Breast and gynecological	2015

### Descriptions of the initiatives

#### Organization format

Studies were conducted in 17 countries: France, Belgium, Switzerland, Ireland, Sweden, Iceland, Finland, Denmark, Norway, England, Luxembourg, Spain, the USA, Italy, Canada, New Zealand, and Australia. Most initiatives (15/18) were housed within one or multiple hospitals or clinics [29–43]. Eleven programs were initiated by an independent organization and 7 by a hospital [32, 36, 38, 39, 41, 42, 44]. Services of 3 initiatives were (additionally) provided in the patients’ home settings [29, 32, 45].

#### Selection and training of volunteers

Six programs solely recruited volunteers who had personal experiences with cancer [30, 34, 38, 40, 41, 43]; in 5 of these programs, patients were paired with volunteers who had been diagnosed with a similar cancer diagnosis [30, 38, 40, 41, 43]. Because of the pairing, volunteers were only women in 4 studies [30, 38, 40, 43]. All but one study reported that volunteers with personal experiences were recruited at least one year after their cancer treatment. One study exclusively recruited volunteers without a personal cancer experience [32] and other studies (7/18) recruited volunteers irrespective of a personal cancer experience [31, 33, 35–37, 39, 45]. The remaining 4 studies did not mention on what basis they recruited volunteers [29, 42, 44, 46].

All but 3 studies [39, 42, 46] indicated that volunteers required some preparation before they were allowed to provide informal care: 12 studies described how volunteers had to follow a training program, another reported that new volunteers reviewed a detailed checklist with a trained volunteer and were required to take part in the unit’s weekly conferences [31], and 2 studies mentioned that the volunteers were instructed but did not describe what kind of instructions were given [41, 43]. The duration of the training varied from 8 to 20 h and training programs differed. Some were thoroughly described and covered medical, emotional, communicational, professional, and ethical aspects of working with patients with cancer [32, 35–38, 42, 45]. Five studies did not clearly mention the content of the training programs [29, 30, 33, 34, 40]. Additional assistance in terms of monthly follow-up meetings or the guidance of volunteer coordinators or experienced volunteers was mentioned in ten studies [31–38, 40, 45].

#### Care provided

In most studies, the care provided consisted of emotional support (15/18) [31–34, 36–46] and/or providing information to patients and their families (13/18) [29, 30, 32, 33, 36–44]. In addition, 6 studies offered occupational and recreational therapy such as relaxation techniques, the cosmetic course “Look Good Feel Better,” painting, singing, and bingo games [29, 31–33, 35, 39, 42]. Some studies (11/18) mentioned practical services such as assisting or transporting patients to the doctors’ offices and/or helping to coordinate their care with medical staff [29, 32, 33, 36–39, 46], visiting the patient in the hospital [30, 33, 38, 43], providing care in the patients’ home settings [29, 32, 45], giving direct financial assistance through a patient support fund [38], and providing a temporary lightweight prosthesis [30]. Seven studies paired volunteers with patients or upon patients’ requests, and one-to-one care was provided [30, 33, 38, 40, 41, 43, 46].

### Evaluation

#### Outcomes

The studies included differed regarding the evaluation of outcomes and no similar outcome measures were used. Only one study compared the experiences of patients who used the program to patients who did not [33]. Fifteen studies focused on experiences and satisfaction levels of patients, family, volunteers, and/or nurses [32–46]. There was a wide variation in how thoroughly the data collection was performed, as well as the description thereof. Most studies surveyed participants and used open-ended questions (9/18) [33, 36–39, 41, 43, 44, 46], questions about demographics (11/18) [32, 33, 36–39, 41–44, 46], and/or self-designed questionnaires (8/18) [32, 33, 38, 40, 42–44, 46]. Three studies did not clearly state how information was obtained [34, 35, 45], of which, just one study mentioned that qualitative information was collected with the help of interviews, but did not mention which questions were asked [34]. Another study exclusively gave statements and quoted patients and volunteers [35]. Finally, one study simply reported that the volunteers’ experiences were assessed at their monthly support meetings but gave no information on how the patients’ experiences were gathered [45].

The remaining 3 studies were descriptive in nature: they described the collaboration between formal and informal supportive care and details on the care provided, but no outcomes were measured [29–31]. Results and outcomes of the articles included are summarized in Table 2.

**Table 2:** Overview of outcomes from 18 studies

Reference	Collaboration	Professions involved	Setting	Care provided	Outcomes
Miller [29]	The American Cancer Society	Volunteers recruited by the American Cancer Society	Services provided by the volunteers of the American Cancer Society in healthcare settings in the USA	Services such as information and counseling services whereby qualified volunteers are trained to answer general questions on cancer asked by patients, but do not give specific medical professional advice, hospitalization, encouraging the development of necessary facilities within a community for the care of patients with cancer in hospitals and other facilities, occupational and recreational therapy, rehabilitation, assisting in the process of restoration of the patient as a functioning social and economic unit (preferably returning the patient to his original occupation so far as practical), transportation, assisting patients to doctors’ offices, hospital outpatient clinics, referring specific inquiries directly to the family physicians or through County Medical Societies to appropriate physicians within the area, providing home care and home nursing services for medically indigent patients with cancer	-
	The American College of Surgeons, the Cancer Detection Center, the National Cancer Institute. United States of America (USA)	Surgeons, physicians, nurses, social services		Providing care in own profession	
Timothy [30]	The ‘reach to Recovery Program’ of the American Cancer Society	Former mastectomy patients recruited by the American Cancer Society	Services provided by volunteers at the hospital, dedicated to helping the woman who just had breast surgery	Volunteers visit the mastectomy patient in the hospital or the clinic within 3–6 days following her operation, give patients a booklet (summarizing the useful information gathered over the years) and a temporary lightweight prosthesis, and answer non-medical questions of patients	The Reach to Recovery Program is implemented in multiple European countries such as France, Belgium, Switzerland, Ireland, Sweden, Iceland, Finland, Denmark, Norway, England, Luxembourg, Spain
	Hospitals and clinics in USA, France, Belgium, Switzerland, Ireland, Sweden, Iceland, Finland, Denmark, Norway, England, Luxembourg, Spain	Hospital staff		Providing care in own profession	
Witter [31]	Volunteers recruited by the St. Joseph’s Oncology Unit in Houston, USA	Individuals that were prompted by the recruiting efforts, with medical interests, a cancer-related trauma, or a desire to help	Services provided by volunteers in the St. Joseph’s Oncology Unit	Services include: providing support for the patients, the families, and the staff, assisting with meal trays, taking messages from patients, visiting patients, shampooing patients, making beds, running to the pharmacy, and providing recreation and diversion by painting, singing and, running the bingo games	-
	Healthcare professionals of St. Joseph’s Oncology Unit in Houston, USA	Physician, nurse, psychotherapist, laboratory technician, physiotherapist, clergyman, social service worker, dietitian, aide, and occupational therapist		Providing care in own profession	
Garrison [45]	The ‘oncology outreach program’ Junior League of Toledo	Individuals with the following qualifications: mature, compassionate, empathetic, a good listener, well informed regarding general oncology and patient needs, resourceful, reliable, flexible in accepting the values of others, general good health and emotional stability, able to provide own transportation and accept mandatory attendance at the training sessions	Services provided by volunteers of the Junior League of Toledo in the home setting of cancer patients to facilitate a smooth transition from hospital to home	Services such as arranging an in-hospital meeting to explain the program and exchange telephone numbers, making a general assessment of the former patient’s situation at home, checking that stipulated services are being provided, listening empathetically to the patient and family members, verifying that the resources specified in the patient’s record are available	Patients Most patients who have received the service have agreed to continue it when they were readmitted and discharged a second time from the hospital. Patients adjusted to their homes with greater ease and security. Most important was the fact that patients and their families realized that the hospital and community care about them as a whole unit in their environment Volunteers Volunteer job satisfaction was assessed at the volunteers’ monthly support meetings. Volunteers’ evaluation of the program was excellent, and those who have been active participants have found the service they provided to be a rewarding experience
	Flower hospital in Sylvania, USA	Hospital staff, e.g., social workers and volunteer coordinators		Providing care in own profession and train and coordinate volunteers	
Fusco-Karmann [32]	The “Voluntary Service program” of the Italian League Against Cancer	Individuals that had no personal experience with cancer	The “Voluntary Service program” is a program in which volunteers gave their support to patients in consulting rooms and hospital departments or along with teams of home palliative care. In 1994, the group of volunteers consists of 700 units: 240 are active in various oncologic hospital departments or consulting rooms (including two hospices), and 250 follow patients at home. The remaining volunteers work in the area of prevention and fundraising	Services such as: psychosocial support, support for families, help in socializing, help for information, help in transport, external errands, spiritual support, note social and economic problems, diverting activities, help in nourishment, and help in cooking and housekeeping at home	Patients A high quota of patients gave a very positive opinion (‘much’ or ‘very much’) on the importance of the presence of volunteers in the hospital (76%) and at home (90%). In the second case, volunteers were particularly useful to improve a patient's mood (80%) and to solve practical problems (47%). Home volunteers were considered more useful than hospital volunteers in every topic investigated, particularly in enlightening spirit (80% home vs 32% hospital) Volunteers and nurses General agreement was noted among nurses and volunteers on the activity of the latter. To the item psychosocial support, a score of 7 to 10 was given by 60% of the nurses and by 82% of the hospital volunteers. At home, respectively 80% and 89% gave such a score. In the comparison between hospital and home environments, a greater number of nurses and volunteers experienced the benefit of hospital volunteers in giving information and in pointing out problems of social and economic matters, and of home volunteers in giving psychosocial support to patients' families
	Milan National Cancer Institute, Italy	Hospital staff		Providing care in own profession	
Edgar [33]	A voluntary support system called ‘Hope and Cope’, an independent organization	Volunteers, one-third of whom are cancer survivors, while the rest have a family member or friend as a referent	Services provided by volunteers in the Sir Mortimer B. Davis-Jewish General Hospital	Services such as being present during Oncology and Radiation Oncology Clinic hours, companions outside the hospital, includes visits and telephone contact, matching as role-models upon patients’ request, offering Hope and Cope Library Office, transportation to radiotherapy sessions, self-help groups, the cosmetic course ‘Look Good Feel Better’, hospital visiting, bereavement, education such as relaxation techniques and writing a newsletter	All patients and family members (both users and non-users) attending either the oncology out-patient or the radiation oncology clinic were approached. Information about the subjects’ knowledge and use and benefits of Hope and Cope services was measured by a questionnaire on 18 different areas of services in Hope and Cope. Information was elicited about their needs for information, social and emotional support, help with the activities of daily living, help with financial concerns, and assistance with employment issues Patients Ninety patients had used some of the services of Hope and Cope, and 31 had not. Significantly more women than men used Hope and Cope; 80% of the female patients and 50% of the men made use of the services. The results showed that needs for social and emotional support and information were most predominant. The most frequently used services were the volunteers in the oncology and radiotherapy clinics, followed by the library, office volunteers, and Hope and Cope staff. The volunteers’ role was perceived to be that of offering hope, encouragement, understanding, reassurance, and giving information in 86% Family/friends The most used services were volunteers in the radiotherapy and oncology clinics, the library, self-help groups, office volunteers, and volunteers to listen. The needs of family members/friends mirrored those of the patients. The most frequent suggestions for improvement were to visit hospitalized patients as soon as possible after the diagnosis and to help patients learn about services earlier
	Sir Mortimer B. Davis-Jewish General Hospital in Montreal, Canada	Professional staff, e.g., social workers		Hope and Cope staff: train the volunteers, screen referrals and match them with some aspect of voluntary support and provide support for the volunteers	
Jones [44]	The Princess Margaret Hospital (PMH) Patient education program developed by the departments of Psychosocial Oncology, Volunteer Resources, and Patient Education, and Wellspring	Trained volunteers	The PMH patient education program is an interactive website aimed toward empowering those dealing with cancer by providing information, tools, and support. It is a user-driven site supported by trained volunteers, in which users can move around freely and at their own pace and determine what they want to see	Providing computer assistance to users, managing resources, and supporting patients	During a 6-month pre-launch period, a pilot study was conducted to evaluate the usefulness, ease of use, and format of the program, and to identify any potential errors and weaknesses in the design Patients, family members, and oncology professionals Participants were invited to complete a brief questionnaire in which they provided demographic information and rated and commented on the usefulness and format of the program. 47 users completed the questionnaire, an additional group of 28 users participated in interview sessions. The most commonly identified user problem was confusion during navigation through the program. Users lacking computer and cancer literacy found the assistance of volunteers to be essential for effective navigation. Volunteer support helped them to access the information they ‘would not have accessed otherwise’ and is clearly an essential instructional strategy for the program
	The PMH of the University Network in Toronto, Canada	Healthcare professionals		Recruiting and training volunteers and providing care in own profession	
Burton [34]	The volunteer program of The Cancer Council NSW’s Breast Cancer Support Service (BCSS)	Volunteers who have had a diagnosis of breast cancer	The volunteer program harnesses the positive experiences and recovery of volunteers to assist people newly diagnosed with breast cancer	Providing peer support and visiting patients after women had surgery	Volunteers Almost all volunteers agreed that most visits they made were positive and rewarding and that they were able to help women by sharing experiences. Volunteers gained satisfaction in being able to ‘give something back’ after their own experience of breast cancer and felt it was rewarding to let other women know they were not alone Coordinators Coordinators expressed high levels of satisfaction in dealing with volunteers and felt supported in their role by The Cancer Council NSW. With regards to improving the service, several coordinators commented about how they had designed better systems to ensure women were told about the BCSS
	Public and private hospitals in several regions in Australia	Women’s health coordinators, palliative care coordinators, Breast screen nurse counselors, stomal therapists, oncology nurse consultants, and cancer care coordinators		Coordinators match volunteers to new referrals as closely as possible by age, treatment type, social setting, and culture where applicable	
Sparks [46]	The ‘Road to Recovery program’ of the American Cancer Society (ACS)	Volunteers, not required to have a prior experience with cancer, volunteer coordinators and ACS staff	An ACS service in which volunteer drivers assist patients with cancer and their families with transportation to treatment facilities and returning them to their homes	Assisting patients with cancer and their families with transportation to treatment facilities	Patients and volunteers were asked questions about experiences, perceived needs, and demographics via a questionnaire, and additionally, some were interviewed Patients Almost all patient respondents (97%) considered Road to Recovery valuable as a means of getting them to their treatment appointments on time, providing them with emotional support, and easing their financial burdens. Many of these patients did express some dissatisfaction about ride availability and the requirement for giving early notice for a ride. Several patients stated that the Road to Recovery program helped to relieve some of the burdens that cancer imposed on members of their families Volunteers The main reasons volunteers gave for volunteering were the desire to help others (48%) and personal experiences with cancer. When asked what volunteers enjoyed most, 80% enjoyed helping patients with cancer, and 19% said that they just liked talking with patients. Almost 8% wanted to help the ACS. Satisfaction with the program was generally high, there were some complaints and the most common suggestion to reduce dissatisfaction was that the ACS should recruit more volunteer drivers
	The mid-Atlantic Division of the ACS and cancer treatment centers and hospitals within the region, USA	Medical providers and social workers		Providing care in own profession and making transportation referrals	
Turner [35]	Healing Partners	The training is open to all health care professionals and to lay individuals	Healing Partners pairs women diagnosed with breast cancer with Healing Touch volunteers who provide free, weekly sessions for six months of energy work, in spaces of Stanford Center for Integrative Medicine, area YMCAs, and medical and bodywork offices Administrative and financial support are also received from Stanford	Healing Touch is a gentle, non-invasive form of energy-balancing work that promotes deep relaxation. It is offered as an adjunct to conventional cancer treatment	Patients Healing Partners participants often cite the experience of deep relaxation as one of the most valuable effects of their Healing Touch sessions. For some participants, the Healing Partners session is one hour each week when they have permission to let go of the stress related to the discomfort, logistics, and uncertainty of their diagnosis and treatment. Some report a reduction of physical symptoms, increased ease of tolerating procedures, and recovery from surgery more rapid than expected Volunteers Volunteers receive great benefits, as well, from their participation in the program. Working with their partner enables them to use their skills in a way that will truly make a difference in someone’s life
	Stanford Center for Integrative Medicine in Palo Alto, USA			-	
Nissim [36]	The Healing Beyond the Body (HBB) program developed by the Department of Psychosocial Oncology and Palliative Care at PMH of the University Health Network in Toronto, Canada	Volunteers, not required to have a prior experience with cancer	A patient-support volunteer program at the Princess Margaret Hospital of the University Network in Toronto Canada	Volunteers provide basic psychosocial support to patients and their families during their hospital visits, facilitate support for non-medical needs, assist in liaison between patients and staff, and facilitate early identification of patients and their informal caregivers who may require professional psychosocial services	Patients Participants were ‘overwhelmingly’ positive about volunteers serving in supportive care roles. They found HBB volunteers more approachable and less busy than clinicians and nurses and easier to access. They perceived that the HBB volunteers were specifically there to support the patients, without having other clinical responsibilities, which meant that they were able to be more responsive to patients’ non-medical needs. The following themes were identified with semi-structured interviews concerning perceived benefits by patients of the HBB volunteer support service: (1) a sense of humanization and normalization; (2) a sense of security; (3) support for nonmedical needs; and (4) support for unaccompanied patients While no negative experiences with the HBB volunteers were reported, potential weaknesses of the HBB volunteer service were identified: (1) a limited awareness by patients of the HBB volunteers’ roles and responsibilities; and (2) the lack of a structured role definition for the HBB volunteers at the pre-treatment phase
	The Princess Margaret Hospital (PMH) of the University Network in Toronto, Canada	Social workers, nurses, and clinicians		Two social workers who are responsible for the screening, training, providing support for volunteers, and supervision of HBB volunteers	
Jasperse [37]	The ‘Living Well Cancer Education and Support Program’ offered by the Cancer Society of New Zealand (CSNZ)	Prospective facilitators of which the majority have a health professional background. Some are former cancer patients. Facilitators can either be volunteers or formally contracted staff members of the CSNZ	The CSNZ provides the program for patients and their supporters in all divisions of New Zealand except Auckland	Providing information on cancer, facilitate informed decision making with respect to disease and side effect management, empower patients to ask the right questions of health professionals and allow patients to gain a measure of control over their situation and maintain that self-efficacy	(Volunteer) facilitators Seventeen facilitators participated in the evaluation. All respondents expressed satisfaction with the content and delivery of the training program. The majority discussed the importance of attending ongoing training. The pairing of more experienced staff and volunteers to co-facilitate was a particularly successful aspect of the program. The majority (12/17) of participants discussed their desire to make a meaningful contribution to their community. The main drawbacks were limited access to support, lack of supervision, and a perceived lack of appreciation from the organization for the volunteer facilitators
	The CSNZ	Employees of the CSNZ: health professionals such as nurses, social workers, and field officers		Pair up with volunteer facilitators, provide care in own profession	
Moulton [38]	‘Woman to Woman’ (WtW) is a peer to peer Gynecologic Oncology Support Program initiated by a survivor of ovarian cancer, the Department of Obstetrics, Gynecology and Reproductive Science, and the Department of Social Work Services of the Mount Sinai Hospital in New York City, USA	Survivors of gynecologic cancers	A professionally-led, peer to peer support program for women with gynecologic cancer at The Mount Sinai Hospital	WtW is committed to addressing the psychosocial needs of women with gynecologic cancers and their families. It provides service such as: giving information and crucial resources on treatment, providing empathic support, following up contact with patients with phone calls or e-mails during treatment, following patients to whom they are matched throughout treatment, visiting them in the inpatient floors, outpatient chemotherapy and radiation centers and at the gynecologic oncology outpatient clinic, system navigation, coordinate care with professional staff, providing practical information on hospital organization, accompanying patients to doctor’s appointments and diagnostic testing, financial support for out of pocket expenses incurred during treatment, including transportation costs, payment of treatment-related bills, wigs, and other treatment-related needs (WtW Patient Fund), planning and implementing educational conferences, public speaking and writing a bi-annual newsletter	Patients Women were telephone surveyed about their experiences with WtW and their perceptions of the effectiveness of the program, strengths, and weaknesses of the program, and how the program met their psychosocial needs The results suggest that the WtW program helped the majority of women cope emotionally with the new cancer diagnosis (98%) and treatment (96%) and helped manage anxiety and fear about receiving a cancer diagnosis (96%). The volunteers provided hope for the possibility of a positive treatment outcome (98%), a needed source of additional support and understanding through their shared cancer experiences (95%), and practical advice and suggestions about managing the physical side effects of treatment (93%). The WtW program also decreased feelings of being alone while facing the cancer experience (93%). Fewer respondents, however, found that WtW helped with communication with a partner (42%), friends or relatives (66%) regarding diagnosis and treatment, helped with supporting the women’s own care network (60%), or provided financial resources (28%)
	The Mount Sinai Hospital in New York City, USA	Medical and social work staff with experience in gynecologic cancer		Screen volunteers and provide monthly meetings to monitor the emotional health of the volunteers, counsel patients when the volunteer is unable to continue her work, and raise additional funds	
Lorhan [39]	The Volunteer Services Department of BC Cancer Agency, Vancouver Island Centre (BCCA-VIC), Canada	Volunteers	Volunteers of the BCCA-VIC lay navigation program at the BC Cancer Agency, Vancouver Island Centre to provide patients undergoing treatment. Volunteers work in concert with the staff team to meet the emotional-, practical-, and informational needs of patients	Providing direct support to patients. The responsibilities of these positions included greeting patients, assisting with navigation through the center, offering companionship in waiting rooms and during treatment, assisting with patient education, and offering therapeutic touch and relaxation therapy	Patients’ demographics, interviews with cancer patients currently undergoing treatment, and three focus groups were used. Patients, volunteers, and staff all agreed that volunteers enhance the patient experience, specifically by working with professionals to meet patients’ emotional, practical, and informational needs. Physical needs were indirectly met by guiding patients to the appropriate personnel Patients Companionship helped to ease patient anxiety and provided a social connection. Patients were able to obtain the information they were looking for through staff and volunteers at the cancer center Practical support was identified as a key benefit of volunteer support in patient interviews and all three focus groups as they would not have to worry about practical issues as the volunteers would take care of them Volunteers and staff Volunteers noted that there was a sense of comfort and safety that the patients experienced. Volunteers noted that they often needed to spend time with patients reviewing the information they received and pointing out available services. Staff emphasized the importance of volunteers easing the stress of patients while they waited in the waiting room; the volunteers eased anxiety by giving patients personal attention
	BC Cancer Agency, Vancouver Island Centre (BCCA-VIC), Canada	Community representatives and BCCA-VIC healthcare staff members: community nurse manager, clinic manager, clerk, radiation therapist, counselor, dietician, and a clinic nurse		Providing care in own profession	
Loprinzi Brauer [40]	The ‘Pink Ribbon Mentorship Program’ of the Mayo Volunteer Services	Breast cancer survivors	A one-on-one peer mentorship program in the Mayo Clinic Comprehensive Cancer Center to provide support to newly diagnosed breast cancer patients	Volunteers that are called ‘mentors’ provide emotional and informational support to individual patients and their caregivers They work closely with health care providers to provide comprehensive support to newly diagnosed breast cancer patients during all phases of their care and survivorship	Patients The survey assessed patient perceptions of the program and consisted of items to rate the satisfaction of the breast cancer patients and mentors. They found that the patients had an overall positive experience and were quite satisfied with the one-on-one peer support program. The majority of patients reported that they received support from the mentor, related well to their mentor, that their mentor affected their breast cancer journey in a positive way, and that they were comfortable discussing their cancer with their mentor Volunteers Approximately one-third of the mentors indicated that being a mentor brought up distressing thoughts about their own cancer. However, the results from the survey also show that mentors are very satisfied with their participation in the mentorship program and that the mentors believe that they benefited from participation
	The Mayo Clinic Comprehensive Cancer Center Rochester, USA	Health care providers		The mentors are trained by, and function under, Mayo Volunteer Services	
Borregaard [41]	An initiative by the Cardiothoracic and Vascular surgery department of the Odense University Hospital	A former lung cancer patient	An initiative from the Odense University Hospital with the aim of providing an opportunity for admitted lung cancer patients to exchange experiences with a former patient once every week	Providing emotional and informational support by sharing experiences, problems, and thoughts with patients, and answering non-medical questions	Patients Demographics and answers to open-ended questions were obtained on nine patients. Patients experienced that exchanging emotional thoughts was easier with a peer, talking to a peer reduced loneliness and patients felt that they were the main person in the conversation with a peer. Sharing stories about having similar symptoms and undergoing similar journeys predominated, and the key feature of the contact between patients was the commonality of their stories Volunteer The volunteer stated that the positive impact worked both ways. He benefited by feeling that his contribution had been of some significance to the patients and, in that sense, it worked both ways
	The Odense University Hospital, Denmark	Health care professionals such as nurses and doctors		The nurses informed the patients about the former patient’s disease and also that he was not a member of staff and thereby was able to discuss issues other than those related to professional health care. Posters were put up around the department to inform patients about the initiative	
Ervik [42]	A cancer support center, known as Vardesenter (VS)	Volunteers, one cancer nurse and one assistant	A Vardesenter where visitors may receive information, support, and counseling, take part in activities, meet peers or just find some peace and rest, at the UNN	-	Visiting cancer patients and relatives were asked questions about demographics and experiences, reasons, and expectations for attending the Vardesenter Patients The majority of visitors to the VS were women, and breast cancer was the most frequent diagnosis. ‘To meet others in the same situation’ was the most frequent reason for visiting the center. Visitors wanted better access to peers with a cancer diagnosis, a nurse specialized in cancer care, an oncologist, or volunteers. Four out of five people were very satisfied with their visit to the center and visitors regarded the center both as an integrated and a complementary part of the healthcare system and wanted a cancer care center to be established in their local community
	The University Hospital of North Norway (UNN) in collaboration with the Norwegian Cancer Society, Norway	Health care professionals such as nurses and doctors		Providing care in own profession	
Pitcher [43]	Bridge of Support (BoS) program offered by Counterpart, a state-wide Service in Victoria, Australia	Peer support volunteers, who have themselves experienced breast or gynecological cancer, and BoS program coordinators	The BoS program provides services at the Sunshine Hospital	Providing supportive care and referring to current evidence-based information to assist them with their decision-making. Program coordinators liaise with health service staff and the peer support volunteers to connect women with a volunteer	The perspectives and experiences of women, volunteers, and health service staff were measured by collecting and analyzing program users’ demographic and service use data, and self-administered questionnaires Patients Most women reported positive experiences with the volunteers, including that it was helpful to have someone to talk to (91.8%), especially someone who had been through what they were going through (89.8%), and that volunteers helped to increase their understanding of what to expect in terms of treatment and side effects (79.6%). Women found their contacts with volunteers particularly useful in terms of giving them hope, knowing there was someone who understood what they were experiencing and with whom they could share their experiences, and having someone to listen to them Volunteers All volunteers believed that women benefitted from the information they were able to give them and sharing their experiences. They had received adequate training to undertake their role. None of the volunteers reported that being in the hospital environment was confronting or made it difficult to provide peer support to women Health service staff Most staff reported that they were confident referring women to a peer support volunteer at the hospital and did not believe there were any barriers to referring women
	The Western Health’s Sunshine Hospital in Melbourne, Victoria	Health service staff		Providing care in own profession	

#### Patients’ experiences

Twelve studies evaluated the experiences and perceptions of patients with cancer concerning the care given by the initiatives. All studies found that patients had positive experiences with the programs as they helped the majority of patients to cope with cancer in terms of informational needs, social and emotional support, help with the activities of daily living, help with financial concerns, and family-related issues [32, 33, 35, 36, 38, 40–46].

As mentioned above, only one study made use of a comparison group by including all patients and family members (both users and non-users) attending either the oncology out-patient or the radiation oncology clinic. Differences were found in the needs of non-users compared to users: non-users had less need for information, were less likely to have financial needs compared to users, were half as likely to be in need of emotional support, and had the same few needs for help with daily activities or transportation [33].

Additionally, one study evaluated the program both in a hospital setting and at the patients’ homes and found that patients considered volunteers more useful in the home setting on every topic investigated [32].

Five studies focused only on experiences of patients with specific cancer types such as lung cancer, gynecologic cancer, and breast cancer. These programs found positive results on more specified care such as: providing support in self-image issues related to cancer treatment (e.g., hair loss) [38] and providing a link with local communities of cancer survivors with a similar diagnosis of breast cancer [40].

Two studies focused on weaknesses of the programs and the findings were in line with each other [33, 36]. Both studies found that patients had limited awareness of the volunteers’ roles and responsibilities. Additionally, both studies stated that patients wished to learn about the services earlier: one study explicitly mentioned that patients wanted timely access to information about services to meet their evolving needs (especially at the pre-treatment phase) [36], the other study reported that patients preferred a visit as soon after diagnosis as possible [33].

Some studies explored whether patients felt that a personal cancer experience was a requirement for volunteers. One study found that the majority of patients felt that this was not required as other qualities of volunteers were more important, e.g., showing compassion, empathy, and having a friendly, outgoing nature [36]. This was in line with the findings of another study that found beneficial qualities of volunteers, mentioned by patients during the interviews. These included more “generic” qualities such as good listening skills, being helpful, being resourceful, and giving understanding, and reassurance through interactions with the patients. In this study, personal experiences with cancer were not explicitly mentioned by patients, volunteers, and staff members [39]. On the contrary, in a program that only included volunteers with a personal cancer experience of providing care, patients stated that “it meant a lot to talk to a person who knows how you feel.” Remarks were made to the effect that it felt more appropriate to talk to former patients because they had been through a similar process and thus knew what the patients were talking about [41].

#### Volunteers’ experiences

Ten studies evaluated the experiences of volunteers. All of these found that volunteers had positive experiences with providing care and that volunteers benefited as their contributions were of significance to the patients [32, 34, 35, 37, 39–41, 43, 45, 46]. Four studies that evaluated the experiences of health staff members on the volunteers’ care provided were in line with these findings, and agreed that volunteers enhanced the patient experience, specifically by working together with professionals to meet the patients’ emotional, practical, and informational needs [32, 39, 43, 44]. In one of these studies, both nurses and volunteers were asked to fill in a questionnaire on the contributions of the volunteers. Volunteers and nurses largely agreed on, e.g., providing help for information, support for families, and spiritual support. However, volunteers considered themselves more engaged in the psychosocial support of patients rather than in supporting, relaxing, and comforting activities, in contrast to the opinion of nurses [32].

Some remarks and negative experiences were reported in 4 studies [34, 37, 40, 46]. One study found a lack of supervision, lack of communication on the training program, and lack of valued appreciation of their work by their volunteer facilitators [37]. A different study made suggestions on how to improve the services. For example, volunteers wished for further training to be able to respond to patients’ requests for information on a broad range of topics. Volunteer coordinators commented on developing a protocol with the local nursing unit manager to ensure that patients were told about the services [34]. In one study measures, e.g., car insurance coverage and reimbursement for gas, and recognition for the services provided, were reported as reducing volunteers’ dissatisfaction in providing transportation services [46]. Furthermore, one study evaluated the experience of trained volunteer breast cancer survivors, who provided care at least one year after cancer treatment. This study found that one-third of the volunteers reported that the service brought up distressing thoughts about their own cancer experiences. Hence, the study stated that support for the volunteers was a crucial component of a successful program. On a positive note, the study reported that volunteers were very satisfied with their participation in the program, and that the volunteers believed that they benefited from participation in monthly mentor meetings. These meetings provided education and support to help volunteers manage distressing feelings regarding their own breast cancer diagnosis: they obtained support and learned additional coping skills to manage their distress [40]. By contrast, another study reported that none of the volunteers felt that being in the hospital environment was confrontational or made it difficult to provide peer support to women, nor that they felt ill-equipped to deal with women’s concerns, or that volunteering was more challenging than they had anticipated [43].

## Discussion

This study aimed to learn more about (1) which types of initiatives that combine formal and complementary informal supportive care for patients with cancer are described in the literature, (2) what type of care they offer, and (3) how they are evaluated in terms of outcome measures. Most initiatives (15/18) were housed within one or multiple hospitals or clinics, and 11 programs were initiated by an independent organization and 7 by a hospital.

The satisfaction with the care offered by these initiatives was evaluated in patients, volunteers, and hospital staff, and related to informational needs, social and emotional support, help with activities of daily living, help with financial concerns, and/or issues in the home environment. Even though there was high heterogeneity in the type of care the initiatives provided, all care was evaluated positively. Some remarks were made by patients on the limited awareness of the services, and the volunteers’ roles and responsibilities.

We found that the quality of the studies included varied greatly and studies were very diverse in terms of the outcome measures used for evaluation. Hence, it was not possible to combine the outcomes into a formal meta-analysis. This lack of similarity in the outcome measures may have related to a lack of consensus on the precise goal of the initiatives, other than “providing support to the patient.” In addition, it is difficult to perform studies on the effectiveness of initiatives which are already implemented as this may lead to the withholding of care from subgroups of patients. However, it should be possible to investigate differences in effectiveness between hospitals, if there is variation in the way they have organized the combination of formal and informal care, and if one is able to control for differences in patient populations and other circumstances. This in turn might be complicated, and to date, no comparison between different centers has been undertaken. However, it is worth noting that studies focusing on the effectiveness of experimental interventions showed promising results in terms of informational needs [47, 48], reduced stress, increased hope, and overcoming loneliness [47–50] in patients with cancer. For example, Schofield [51] compared an intervention, in which formal and informal cares were combined, to care as usual in women with gynecological cancers receiving curative radiotherapy. The nurse- and peer-led psycho-educational intervention consisted of nurse-led consultations and peer telephone support, and patients allocated to usual care received a cancer council booklet and information from their treatment team. Although no effects were found for psychological distress, patients in the intervention arm had better treatment readiness, fewer needs for information on the health system (e.g., procedural concerns), and fewer concerns about sexuality. Future research, in the form of high-quality controlled trials, is warranted to investigate whether the combination of formal and informal care is of additional value for patients with cancer in terms of information needs and supportive care compared to solely receiving care as usual.

As shown in this review, nearly all studies (17/18) included only patients who found their way to the initiatives and it may be that these “users” were positively biased compared to “non-users.” On the other hand, the initiatives were not meant to be compulsory, and there is no reason to believe that all patients and/or relatives were in need of the care offered by these initiatives. In that respect, the investigations were not biased, because they focused on the intended target populations. There was limited information given on patient characteristics, e.g., socioeconomic status. What was reported was that more women than men made use of the services. This, however, does not mean that an investigation among “non-users” is without utility. Such an investigation could provide insights into the needs of ‘non-users’ on information and support, which may be accessed through a different channel, such as formal care or web-based programs. One can even suggest that it is not only the patient who is the “consumer” of the benefits, but that there are different stakeholders who also experience benefits. For example, the physician might benefit from more well-informed and compliant patients. And if not only patient satisfaction is increased but also the time and energy of the physician is better utilized, the initiatives may hold the promise of reducing the demands on the healthcare system.

As a result of focusing on (nearly) implemented programs, we evaluated only initiatives/programs that succeeded. Some programs may have failed for a variety of reasons. However, to our knowledge, no studies describing the failure of implementation have been reported in the literature to date. It would be interesting to gain an insight into these reasons, as this could help to prevent failure in launching future programs.

Several studies focused on the volunteers’ experiences and the benefits of their participation. Volunteers stated that most visits they made were experienced as positive and rewarding and they were confident about their contribution to general healthcare. Some volunteers commented on the need for further training as many patients asked for information about a broad range of issues. All but 3 studies reported that volunteers followed some sort of preparation before they were allowed to provide care. Thus, there appears to be a consensus that preparation, in terms of training programs or instructions, is a necessary condition for volunteers to provide care successfully. A variation in the recruitment of volunteers was observed within the studies. Three studies focused on whether patients felt that a personal experience with cancer was a requirement for volunteers. Two studies found that the majority of patients felt that this was not required as different qualities were more important. By contrast, in one study, only volunteers with a personal cancer experiences provided care, and patients stated that “it meant a lot to talk to a person who knows how you feel.” No conclusion can be drawn yet as to whether it would be beneficial for the care provided if volunteers have personal experiences with cancer.

Based on the literature search we believe that the combination of formal and informal care at least holds the potential of added value, since informal care is trained by, and can refer to, formal care. The quality of the initiatives can be guaranteed as healthcare professionals provide feedback on the services of these programs/initiatives. Moreover, this combination has the greatest potential to be cost-effective, since it can probably substitute formal care more easily than when informal care is delivered independently of formal care. It can be hypothesized that informal care provided by volunteers is relatively inexpensive compared to formal care requiring provision by healthcare professionals. Unfortunately, none of the articles examined provided information on costs. A closer look at the quantity and quality of the services these initiatives provide, and the related costs, could help to provide insights into their cost-effectiveness. It would thus be interesting to gain more insights into who benefits from these initiatives in addition to patients, and into their cost-effectiveness.

## Conclusion

In summary, evaluating initiatives that combine both formal and informal supportive care is difficult since consensus on goals and outcomes is lacking. This does not mean that the outcome of this review should be regarded inconclusive. If the goal of such initiatives is patient satisfaction with the care provided, such as information and/or support, then, the outcome of this review is positive. Based on the review’s results, initiatives combining formal and informal supportive care at least hold the potential of added value for both patients with cancer and their families in coping with the diagnosis, treatment, and consequences thereof. In this respect, support for, and adequate training of, volunteers can be viewed as success factors.

## Supplementary Information

Below is the link to the electronic supplementary material.

Supplementary file1 (DOCX 21 KB)

## Data Availability

The manuscript has no associated data.
