# Supplementary material for: Evaluation of centers for information and support combining formal and informal care for patients with cancer: a systematic review of the literature
Source: Support Care Cancer. 2022 Apr 13;30(9):7079–98. doi: 10.1007/s00520-022-07047-w (PMC9385827; doi:10.1007/s00520-022-07047-w)
Supplement: Supplementary file 1 — Supplementary file1 (DOCX 21 KB) [file 520_2022_7047_MOESM1_ESM.docx]

# Supplemental files

### Supplementary Table 1

Supplementary Table 1: full search strategy and results until July 2019, 2021

| **Embase.com** | ('volunteer'/mj OR 'hospital volunteer'/de OR 'voluntary worker'/de OR ('peer group'/de AND ('caregiver'/de)) OR ('organization and management'/de AND ('health care personnel'/exp)) OR 'personnel management'/de OR (((volunteer* OR voluntar*) NEAR/6 (care OR carer* OR caregiver* OR hospital* OR palliat* OR hospice* OR role OR service* OR team* OR assist* OR network* OR peer* OR involve* OR worker* OR employee*)) OR (volunteer* NEXT/3 program*) OR (peer NEXT/1 (care OR carer* OR caregiver*)) OR peer*-to-peer* OR voluntaris* OR ((personnel* OR human-resource* OR professional*) NEAR/3 (management* OR organization* OR organisation*))):ab,ti OR volunteer*:ti) AND ('oncology'/exp OR 'neoplasm'/exp OR 'cancer patient'/exp OR 'cancer surgery'/exp OR 'cancer survival'/exp OR 'cancer survivor'/exp OR chemotherapy/exp OR 'palliative therapy'/exp OR 'mastectomy'/exp OR 'cancer center'/de OR 'cancer therapy'/exp OR 'cancer pain'/exp OR 'oncology ward'/exp OR 'immunotherapy'/exp OR (oncolog* OR neoplasm* OR cancer* OR tumor OR tumour* OR mastectom* OR chemotherap* OR chemoradiotherap* OR radiochemotherap* OR immunotherap*):ab,ti) AND ('mental function'/exp OR 'psychology'/exp OR 'social care'/exp OR 'patient satisfaction'/de OR 'social medicine'/exp OR (mental* OR psycholog* OR social* OR psychosocial* OR support* OR (patient* NEAR/3 (satisf* OR experience*))):ab,ti) NOT (((healthy NEXT/2 volunteer*)):ab,ti OR (euthanasia* OR suicide OR assisted-dying OR assisted-death):ti) NOT ([Conference Abstract]/lim AND [1800-2017]/py) NOT ((animal/exp OR animal*:de OR nonhuman/de) NOT ('human'/exp)) AND [English]/lim |
| --- | --- |
| **Medline Ovid** | (*Volunteers/ OR Hospital Volunteers/ OR (Peer Group/ AND (Caregivers/)) OR ("Organization and Administration "/ AND (exp Health Personnel/)) OR exp Health Personnel/og OR Personnel Management/ OR (((volunteer* OR voluntar*) ADJ6 (care OR carer* OR caregiver* OR hospital* OR palliat* OR hospice* OR role OR service* OR team* OR assist* OR network* OR peer* OR involve* OR worker* OR employee*)) OR (volunteer* ADJ3 program*) OR (peer ADJ (care OR carer* OR caregiver*)) OR peer*-to-peer* OR voluntaris* OR ((personnel* OR human-resource* OR professional*) ADJ3 (management* OR organization* OR organisation*))).ab,ti. OR volunteer*.ti.) AND (Medical Oncology/ OR exp Neoplasms/ OR Cancer Survivors/ OR Antineoplastic Combined Chemotherapy Protocols/ OR exp Mastectomy/ OR Cancer Pain/ OR (oncolog* OR neoplasm* OR cancer* OR tumor OR tumour* OR mastectom* OR chemotherap* OR chemoradiotherap* OR radiochemotherap*).ab,ti.) NOT (((healthy ADJ2 volunteer*)).ab,ti. OR (euthanasia* OR suicide OR assisted-dying OR assisted-death).ti.) NOT (news OR congres* OR abstract* OR book* OR chapter* OR dissertation abstract*).pt. NOT ((animal/ OR animal*:de OR nonhuman/) NOT (human/)) AND english.la. |
| **PsychINFO Ovid** | (*Volunteers/ OR (Peers/ AND (Caregivers/)) OR Human Resource Management / OR (((volunteer* OR voluntar*) ADJ6 (care OR carer* OR caregiver* OR hospital* OR palliat* OR hospice* OR role OR service* OR team* OR assist* OR network* OR peer* OR involve* OR worker* OR employee*)) OR (volunteer* ADJ3 program*) OR (peer ADJ (care OR carer* OR caregiver*)) OR peer*-to-peer* OR voluntaris* OR ((personnel* OR human-resource* OR professional*) ADJ3 (management* OR organization* OR organisation*))).ab,ti. OR volunteer*.ti.) AND (Oncology/ OR exp Neoplasms/ OR exp Mastectomy/ OR (oncolog* OR neoplasm* OR cancer* OR tumor OR tumour* OR mastectom* OR chemotherap* OR chemoradiotherap* OR radiochemotherap*).ab,ti.) NOT (((healthy ADJ2 volunteer*)).ab,ti. OR (euthanasia* OR suicide OR assisted-dying OR assisted-death).ti.) NOT (news OR congres* OR abstract* OR book* OR chapter* OR dissertation abstract*).pt. AND english.la. |
| **CINAHL EBSCOhost** | MH Volunteer Workers OR (MH Peer Group AND (MH Caregivers)) OR TI(((volunteer* OR voluntar*) N5 (care OR carer* OR caregiver* OR hospital* OR palliat* OR hospice* OR role OR service* OR team* OR assist* OR network* OR peer* OR involve* OR worker* OR employee*)) OR (volunteer* N2 program*) OR (peer N1 (care OR carer* OR caregiver*)) OR peer*-to-peer* OR voluntaris*) OR AB(((volunteer* OR voluntar*) N5 (care OR carer* OR caregiver* OR hospital* OR palliat* OR hospice* OR role OR service* OR team* OR assist* OR network* OR peer* OR involve* OR worker* OR employee*)) OR (volunteer* N2 program*) OR (peer N1 (care OR carer* OR caregiver*)) OR peer*-to-peer* OR voluntaris*) OR TI(volunteer*)) AND (MH Oncology+ OR MH Neoplasms+ OR MH Cancer Survivors OR MH Chemotherapy, Cancer+ OR MH Mastectomy OR MH Cancer Pain OR (TI(oncolog* OR neoplasm* OR cancer* OR tumor* OR tumour* OR mastectom* OR chemotherap* OR chemoradiotherap* OR  radiochemotherap*)) OR AB (oncolog* OR neoplasm* OR cancer* OR tumor* OR tumour* OR mastectom* OR chemotherap* OR chemoradiotherap* OR radiochemotherap*))) AND (MH Psychology+ OR MH Patient Satisfaction OR TI(mental* OR psycholog* OR social* OR psychosocial* OR support* OR (patient* N2 (satisf* OR experience*))) OR AB(mental* OR psycholog* OR social* OR psychosocial* OR support* OR (patient* N2 (satisf* OR experience*)))) NOT TI(((healthy N2 volunteer*)) OR (euthanasia* OR suicide OR assisted-dying OR assisted-death)) NOT PT (news OR congres* OR abstract* OR book* OR chapter* OR dissertation abstract*) NOT ((MH animal+) NOT (MH human+)) AND LA(English)  (MM Volunteer Workers OR (MH Peer Group AND (MH Caregivers)) OR (MH "Management" AND (MH Health Personnel+)) OR MH Personnel Management OR TI(((volunteer* OR voluntar*) N5 (care OR carer* OR caregiver* OR hospital* OR palliat* OR hospice* OR role OR service* OR team* OR assist* OR network* OR peer* OR involve* OR worker* OR employee*)) OR (volunteer* N2 program*) OR (peer N1 (care OR carer* OR caregiver*)) OR peer*-to-peer* OR voluntaris* OR ((personnel* OR human-resource* OR professional*) N2 (management* OR organization* OR organisation*))) OR AB(((volunteer* OR voluntar*) N5 (care OR carer* OR caregiver* OR hospital* OR palliat* OR hospice* OR role OR service* OR team* OR assist* OR network* OR peer* OR involve* OR worker* OR employee*)) OR (volunteer* N2 program*) OR (peer N1 (care OR carer* OR caregiver*)) OR peer*-to-peer* OR voluntaris* OR ((personnel* OR human-resource* OR professional*) N2 (management* OR organization* OR organisation*))) OR TI(volunteer*)) AND (MH Oncology+ OR MH Neoplasms+ OR MH Cancer Survivors OR MH Chemotherapy, Cancer+ OR MH Mastectomy OR MH Cancer Pain OR TI(oncolog* OR neoplasm* OR cancer* OR tumor OR tumour* OR mastectom* OR chemotherap* OR chemoradiotherap* OR radiochemotherap*) OR AB(oncolog* OR neoplasm* OR cancer* OR tumor OR tumour* OR mastectom* OR chemotherap* OR chemoradiotherap* OR radiochemotherap*)) NOT TI(((healthy N2 volunteer*)) OR (euthanasia* OR suicide OR assisted-dying OR assisted-death)) NOT PT (news OR congres* OR abstract* OR book* OR chapter* OR dissertation abstract*) NOT ((MH animal+) NOT (MH human+)) AND LA(English) |

### Supplementary Table 2

Supplementary Table 2 – Risk of bias for qualitative studies using the CASP checklist

|  | Section A | | | | | | Section B | | |
| --- | --- | --- | --- | --- | --- | --- | --- | --- | --- |
|  | Q1 | Q2 | Q3 | Q4 | Q5 | Q6 | Q7 | Q8 | Q9 |
| Garrison, 1983 | Yes | Yes | No | No | No | No | No | No | No |
| Jones, 2001 | Yes | Yes | Yes | No | Yes | No | No | Yes | Yes |
| Burton, 2001 | Yes | Yes | Yes | Yes | Yes | No | No | No | Yes |
| Sparks, 2001 | Yes | Yes | Yes | Yes | Yes | No | No | Yes | No |
| Turner, 2005 | No | No | No | No | No | No | No | No | No |
| Nissim, 2009 | Yes | Yes | Yes | Yes | Yes | Yes | Yes | Yes | Yes |
| Jasperse, 2012 | Yes | Yes | Yes | Yes | Yes | Yes | Yes | Yes | Yes |
| Moulton, 2013 | Yes | Yes | Yes | Yes | Yes | No | Yes | Yes | Yes |
| Lorhan, 2015 | Yes | Yes | Yes | Yes | Yes | Yes | Yes | Yes | Yes |
| Borregaard, 2017 | Yes | Yes | Yes | Yes | Yes | Yes | Yes | Yes | Yes |
|  |  |  |  |  |  |  |  |  |  |
|  | Section A: Are the results of the study valid?  Q1. Was there a clear statement of the aims of the research?  Q2. Is a qualitative methodology appropriate?  Q3. Was the research design appropriate to address the aims of the research?  Q4. Was the recruitment strategy appropriate to the aims of the research?  Q5. Was the data collected in a way that addressed the research issue?  Q6. Has the relationship between researcher and participants been adequately considered? | | | | | | | | |
|  | Section B: What are the results?  Q7. Have ethical issues been taken into consideration?  Q8. Was the data analysis sufficiently rigorous?  Q9. Is there a clear statement of findings? | | | | | | | | |
